# Defining the origin, evolution, and immune composition of SDH-deficient renal cell carcinoma

**DOI:** 10.1016/j.isci.2022.105389

**Published:** 2022-10-17

**Authors:** Joana B. Neves, Kirsty Roberts, Janani Sivakumaran Nguyen, Soha El Sheikh, My-Anh Tran-Dang, Catherine Horsfield, Faiz Mumtaz, Peter Campbell, Hans Stauss, Maxine G.B. Tran, Thomas Mitchell

**Affiliations:** 1 UCL Division of Surgery and Interventional Science, Royal Free Hospital, London, UK; 2 Specialist Centre for Kidney Cancer, Royal Free Hospital, London, UK; 3 UCL Institute of Immunity & Transplantation, The Pears Building, Pond Street, London, UK; 4 Wellcome Sanger Institute, Wellcome Genome Campus, Hinxton, Cambridge, UK; 5 Department of Histopathology, Royal Free Hospital, London, UK; 6 Guy’s & St Thomas’ National Health Service Trust, Westminster Bridge Road, London, UK; 7 Department of Surgery, University of Cambridge, Cambridge, UK

**Keywords:** Cancer systems biology, Cancer, Genomics

## Abstract

Succinate dehydrogenase (SDH)-deficient renal cell carcinoma represents a rare subtype of hereditary kidney cancer. Clinical diagnosis can be challenging and there is little evidence to guide systemic therapeutic options. We performed genomic profiling of a cohort of tumors through the analysis of whole genomes, transcriptomes, as well as flow cytometry and immunohistochemistry in order to gain a deeper understanding of their molecular biology. We find neutral evolution after early tumor activation with a lack of secondary driver events. We show that these tumors have epithelial derivation, possibly from the macula densa, a specialized paracrine cell of the renal juxtaglomerular apparatus. They subsequently develop into immune excluded tumors. We provide transcriptomic and protein expression evidence of a highly specific tumor marker, PAPPA2. These translational findings have implications for the diagnosis and treatment for this rare tumor subtype.

## Highlights

• Whole genome sequencing indicates early initiation followed by neutral evolution
• Retained transcriptional signals uncovers a putative macula densa cell of origin
• Immunophenotyping demonstrates an immune-excluded microenvironment
• PAPPA2 shows promising utility as a highly specific tumor marker

## Graphical abstract

**Figure:**
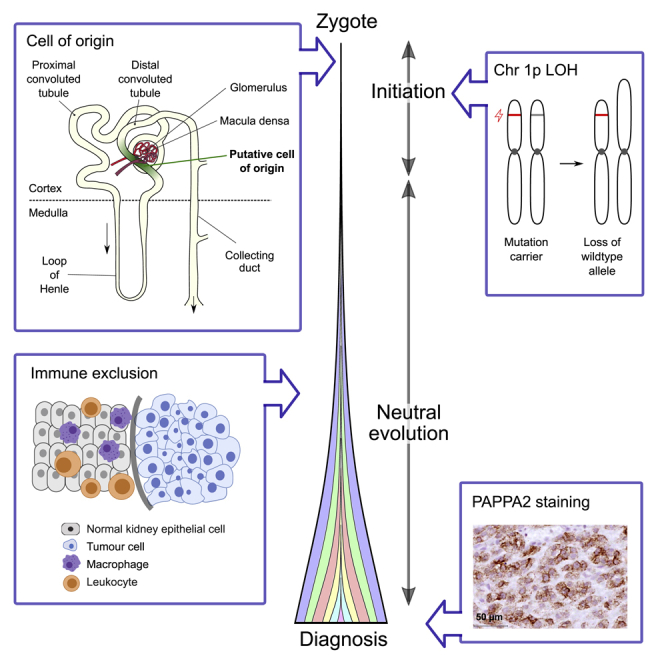

## Introduction

Succinate dehydrogenase (SDH) is a ubiquitously expressed enzyme composed of multiple subunits, including SDHA, SDHB, SDHC, and SDHD.^1^ SDH plays critical steps in the cell’s metabolism, taking part both in Krebs tricarboxylic acid cycle and in the mitochondrial electron transport chain.^2^ SDH catabolizes succinate into fumarate leading to FAD reduction to FADH_2_ and generating electrons that are transported along iron-sulfur clusters on SDHB. There are also two binding sites for ubiquinone (coenzyme Q) on SDH, one between SDHB, SDHC, and SDHD, and another on SDHD. The electrons generated from FAD oxidation then reduce ubiquinone into ubiquinol.

SDH is a tumor suppressor through mechanisms that are largely unknown.^1^^,^^3^ Germline heterozygous mutations in the genes encoding all SDH subunits have been associated with increased incidence of malignancies. Germline carriers are at a higher risk of developing neural crest derived tumors, such as retroperitoneal and head and neck paragangliomas, and pheochromocytomas.^1^ Other less frequently associated tumors include gastrointestinal stromal tumors and renal cell carcinoma (RCC). First reported in 2004,^4^^,^^5^ SDH-deficient RCC has been recognized since 2016 by the WHO as an entity with unique morphologic and genetic features.^6^ SDH-deficient RCC is almost exclusively associated with germline mutations of any of the SDH subunits^7^^,^^8^ but most frequently with mutations in subunit B.^9^ Carriers of SDHB mutations have a lifetime risk of RCC by the age 60 between 4% and 6%.^10^ The population-wide incidence of these tumors is low, corresponding to up to 0.2% of all RCC cases.^8^ Currently, diagnosis is reliant on microscopic pattern,^11^^,^^12^ family history, and/or personal history of germline mutation of an SDH subunit, combined with absent immunohistochemical staining for SDHB.^13^ SDH-deficient RCC has an indolent behavior in up to three-quarters of cases,^8^ but aggressive behavior has also been described,^8^^,^^14^^,^^15^ frequently but not always associated with the presence of high nuclear grade, sarcomatoid differentiation, or coagulative necrosis.^8^

We sought for the first time to thoroughly characterize the molecular features, microenvironment, and evolutionary pattern of a cohort of SDH-deficient RCCs using whole genome sequencing, transcriptomics, flow cytometry, and histology. We provide insights with potential diagnostic and therapeutic implications.

## Results

### Clinical and demographic data

We prospectively collected and analyzed fresh frozen and paraffin-embedded samples of tumor and matched non-affected kidney from four patients with SDH-deficient RCC. All patients had a germline mutation on *SDHB*, with the median patient age at diagnosis of 29 years (Table S1). All tumors were localized and surgically treated, with tumor size ranging from 17 to 130 mm and staging from pT1a to pT2b. Three out of four tumors were unifocal, one patient had an earlier contralateral SDH-deficient RCC surgically excised (PD47453; not sampled). All patients were alive and free of disease at the last follow-up with a median follow-up of 18 months. Multiregional sampling was conducted for one tumor (PD47450) and, for one case, a sample from a synchronously surgically excised paraganglioma was also analyzed (PD47454). The tumor of a fifth patient (PD47452) with suspected SDH-deficient RCC based on immunohistochemical staining was also sampled and analyzed, but the germline and somatic genetic analysis excluded SDH-deficient RCC and confirmed a diagnosis of clear cell RCC (ccRCC). This sample was included in the analyses as a control.

Histopathological examination of the tumors revealed that they were all well circumscribed and displayed the characteristic morphologic features of SDH-deficient RCCs including neoplastic cells with eosinophilic, flocculent cytoplasm, round, and centrally located nuclei with dispersed chromatin, and inconspicuous nucleoli. All tumors displayed cystic change. All tumors had low-grade nuclear features. No evidence of sarcomatoid differentiation or coagulative necrosis was noted.

### Genetic profile reveals neutral evolution and early initiation

**Figure 1:**
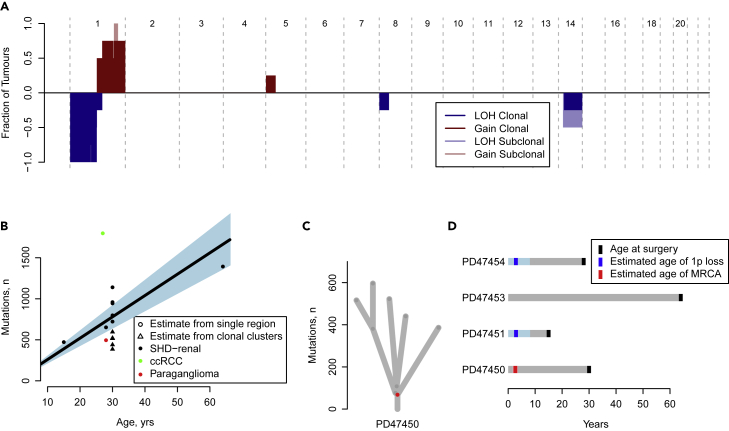
Genomic characterization of SDH-deficient RCCs (A) Merged copy number landscape of the tumors across the genome. Darker colors signify clonal events, lighter subclonal event. Blue denotes loss of heterozygosity, whereas red denotes gains (see also Figure S1). (B) Mutational burden shown with age at sampling. Also shown is the cohort average mutational rate and 95% confidence interval. (C) Reconstruction of the phylogenetic tree for the extensively sampled tumor, PD47450, with the most recent common ancestor shown in red (see also Figure S3). (D) The estimated timing of either loss of chromosome 1p, or emergence of the most recent common ancestor compared to the timing of tissue sampling. 95% confidence intervals are plotted for the estimated age of 1p loss or age of MRCA in lighter colour shades.

Samples were analyzed using whole genome sequencing (mean depth 82x for tumor and 43x for normal). Data were analyzed using validated bioinformatics pipelines to identify somatic copy number alterations, substitutions, insertions/deletions, and rearrangements, and confirm germline SDHB mutations. Sample tumor purity or cancer cell fraction was estimated from results of the copy number analysis (median 81%, range 0.13–0.88, Table S1). The copy number profile revealed that loss of 1p was shared by all analyzed cases (Figure 1A). As *SDHB* is located on 1p, this event removes the wild-type *SDHB* allele to complete loss of function of SDH. In all but one of the tumors, 1p loss was accompanied by gain of 1q. These copy number changes are likely to have occurred simultaneously with the formation of an isochromosome harboring two copies of the long arm of chromosome 1. Typically, the copy number changes occur within the centromeric region, precluding formal confirmation using short-read sequencing technology. There are parallels to the observations of 3p loss and 5q gain in ccRCC,^16^ again representing the initiating event in tumor formation. The sequenced paraganglioma sample exhibited a similar pattern compatible with loss of one copy of the entirety of chromosome 1, followed by whole genome duplication and loss of one copy of chromosomes 3, 11, and 13 (Figure S1).

A median of 728 unique somatic substitutions and 44 indels were identified per tumor (Table S2), with an apparent age dependence. This was formally assessed through the use of a linear mixed effects model (Figure 1B), showing that on average, 26 (SE2.4) mutations were acquired per year of life. We then assessed whether there were any recurrent driver mutations in this cohort. Comparing the ratio of non-synonymous to synonymous mutations,^17^ we found no specific genes under positive selection. The result is unsurprising given the small size of this cohort, and the scarcity of known second driver mutations seen in pheochromocytomas and paragangliomas.^18^ One tumor sample, PD47450e, harbored a truncating mutation in *CDKN2C*, which in addition to the loss of the short arm of chromosome 1 would have resulted in biallelic knockout of this important regulator of cell cycle progression.

Using estimates of tumor purity, copy number state, and the sequencing support of individual somatic mutations, we calculated the cancer cell fraction (CCF), the proportion of cancer cells in each tumor that contained a mutation. We observed in all samples that the majority of called mutations have CCFs below 1 suggesting a high degree of subclonality (Figure S2A). This subclonality was compared to a cohort of previously sequenced ccRCCs.^16^ In the SDH-deficient cohort, a large subclonal fraction with a mean CCF of 0.25 is observed (Figure S2B). In contrast, from the ccRCC cohort, the majority of mutations appear clonal with a peak CCF of 1 with a far smaller subclonal contribution (Figure S2C). We further compared the mutational trinucleotide context of the clonal and subclonal mutations to determine whether either sequencing artifact, contamination, or an alteration in mutational processes might have given rise to the low CCF mutations. No difference was found, indicating that similar processes underpinned the generation of the subclonal and clonal mutations for all tumors sampled (Figure S2D).

We further investigated the phylogenetic structure of PD47450 through the use of multiregional sampling. Each mutation was allocated to a cluster using methods previously described^19^ (Figure S3) to generate a phylogenetic tree (Figure 1C). The phylogeny depicts a short truncal branch, followed by three intermediate nodes, two of which subsequently divide into private branches. The tree displays five terminal branches, one for each geographical area sampled, all of which have an overall similar mutation burden. Taken together, the high subclonality with early branching from the most recent common ancestor supports the possibility of neutral evolution in SDH-deficient RCCs.

We next estimated the time at which the loss event of chromosome 1p occurred. The analysis was restricted to SDH-deficient RCCs with both 1p loss and 1q gain and high purity (PD47450, PD47451, and PD47454). We classified mutations as either pre-duplication or post-duplication based on whether they were present in two of the three 1q copies (i.e. the mutation happened before chromosomal gain) or in only one of the three copies (i.e. the mutation happened after the chromosomal gain). Here, for all samples, the vast majority of the mutations were classified as post-duplication (i.e. present in only one copy of 1q, Figure S4). Assuming a constant rate of mutational acquisition, we used a mixed effects model to estimate the time at which the chromosome 1p loss occurred, as previously described.^16^ In a similar manner, using the number of clonal mutations in the tumor with multiregional sampling, we were able to estimate the chronological time of the most common recent ancestor, i.e. the cell that accumulated all clonal somatic changes immediately prior to subclonal branching within the tumor (Figure 1D). For all samples, the timing of 1p loss and/or the most common recent ancestor was similar and traced back to the first few years of childhood.

### Retained transcriptional signals reveal the cell of origin

**Figure 2:**
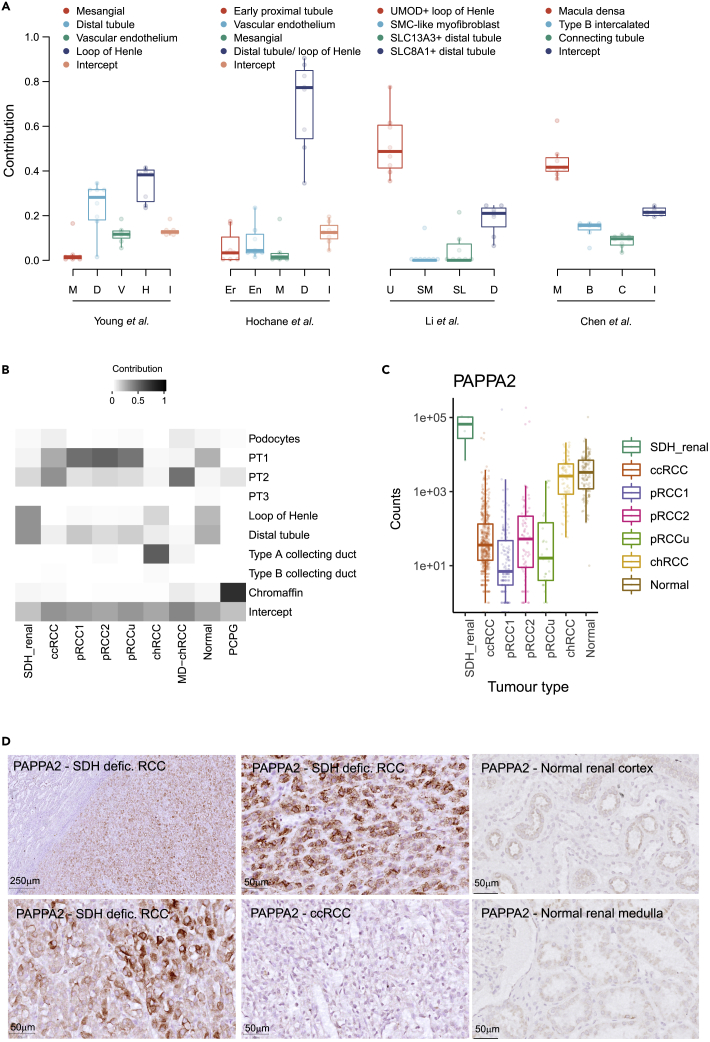
Transcriptional signatures relating to possible cell of origin (A) Cell signal analysis of the bulk RNA transcripts depicted as exposures to different single cell references. Plotting has been restricted to signals that contributed more than an average of 2% and where the maximum exposure was greater than 10% per sample. Reference datasets used from left to right: Adult kidney^21^; fetal kidney^22^; adult kidney^23^; mouse kidney distal nephron.^24^ Plotted are data points for each sample, alongside the median, first and third quartile, and 95% confidence interval of median. (B) Exposures of bulk transcriptomes across multiple tumor types (clear cell RCC (ccRCC), papillary RCC (pRCC) types 1, 2, and unclassified, chromophobe RCC (chRCC), metabolically divergent (MD) chRCC, non-affected kidney (normal), and SDH-deficient paraganglioma and pheochromocytoma (PCPG) RNA data from TCGA), using adult kidney single^21^ and developing adrenal^25^ single cell data as reference. Again plotting has been restricted to signals that contributed more than an average of 2% and where the maximum exposure was greater than 10% per sample. (C) RNA counts of PAPPA2 expression compared to pan-RCC data from TCGA (log2 fold change 9.0, adjusted p value 1.2×10-11). Plotted are data points for each sample, alongside the median, first and third quartile, and 95% confidence interval of median. (D) IHC for PAPPA2 in a selection of three SDH-deficient RCCs, one ccRCC, and one section of normal renal cortex and medulla. The upper left panel contains tumor and normal parenchyma interface.

Using cell signal analysis tools^20^ and single-cell mRNA reference datasets,^21^^,^^22^^,^^23^^,^^24^ we analyzed the bulk tumor transcriptomes for retained patterns that could infer cell of origin. We used four single-cell mRNA reference datasets to allow for a broad and detailed comparison encompassing different anatomic sources (kidney and adrenal), different developmental stages (fetal and adult), and different species (human and mouse) (Figure 2A and Table S3). Firstly, we used a single-cell mRNA reference dataset of adult kidneys.^21^ We observed that RNA contributions were highest for patterns compatible with loop of Henle cells, followed closely by distal nephron cells. Secondly, we used a reference dataset exclusively of human fetal kidney^22^ as the initiating event may have occurred during nephrogenesis. Again, RNA contributions were highest for a pattern compatible with precursor cells to the loop of Henle and distal nephron. The cells from this cluster exhibited high expression of MAL, CLCN5, SLC12A3, POU3F3, SLC12A1, PAPPA2, and UMOD.^22^ Thirdly, we used a further dataset of RCC and non-affected kidney samples from adult patients^23^ with more detailed cellular annotations, to see whether this additional detail could refine the putative cell of origin. Here, RNA contributions were highest for UMOD^+^ loop of Henle cells, but there was also a significant distal tubule contribution. Lastly, in an attempt to resolve the similar observed contributions from loop of Henle and distal tubular cells, and allowing for limitations in the comparison between mouse and human transcriptomes, we used an enriched dataset focused on the distal mouse nephron.^24^ In this comparison, RNA contributions were highest for cells with markers characteristic of the macula densa, a circumscribed and small group of cells that localize within the nephron between the loop of Henle and the distal tubule cells. For this distal mouse nephron reference, contributions from the reference loop of Henle cells were not evident (Table S3).

We next compared the principal single cell-derived signatures from adult kidneys^21^ and adrenal tissue^25^ across the wider spectrum of RCC subtypes, incorporating our data with that from the TCGA^26^ (Figure 2B). As expected, ccRCC and papillary RCCs (pRCC) exhibit a principally proximal tubular signature, whereas chromophobe RCCs (chRCC) exhibit a type A collecting duct signature, and paragangliomas and pheochromocytomas a chromaffin signature. Again, the relative contribution to SDH tumors remains unclear, but clearly appears epithelial in origin, rather than neural crest derived, and distinguished from the other principal RCC subtypes.

We then compared differential expression of single genes between the SDH-deficient RCCs and other RCC subtypes including normal kidney from the TCGA cohort (Table S4). One of the most differentially expressed genes, PAPPA2, was expressed at high levels with a correspondingly low expression in normal kidney and other RCCs (Figure 2C). Pregnancy-associated plasma protein-A2 (PAPPA2) is a metalloproteinase that is responsible for proteolytic cleavage of a subset of insulin-like growth factor (IGF) binding proteins with subsequent release of IGF1, and has been implicated in promoting tumor cell proliferation, invasion, migration, and metastasis in other malignancies.^27^ Non-neoplastic protein expression appears specific to macula densa cells^24^^,^^28^ and the placental extravillous trophoblastic cells.^29^ In the kidney, one of IGF1 actions is to modulate vascular resistance of glomerular arterioles leading to increase in glomerular filtration.^30^ PAPPA2 has been identified in secretory vesicles at the cellular projections extending from the macula densa cells toward the juxtaglomerular apparatus,^28^ suggesting a role in paracrine signals to glomerular structures. Thus, PAPPA2 production by the macula densa could be a putative mediator of glomerular filtration control by modulating local IGF1 availability.

Finally, we sought to determine whether this protein could be used to aid the clinical histopathological diagnosis of these tumors, particularly as loss of SDHB expression is not pathognomonic of SDH-deficient RCC. Here, immunohistochemistry (IHC) for PAPPA2 revealed strong staining among SDH-deficient RCCs, with absence both in normal kidney or representative ccRCC cases (Figure 2D). In addition, PAPPA2 was not consistently expressed in any of the normal cell types annotated from the existing human single-cell RNA sequencing datasets, the likely explanation being the rarity of this cell type in comparison to other cells of the nephron. The only single-cell dataset with clear expression was from murine single-cell transcriptomes enriched from the distal nephron.^24^

### Immunophenotyping reveals a cold tumor microenvironment

The importance of the immune system in regulating tumor behavior is long established. In particular, modulating T cell behavior through immune checkpoint inhibition has increasing therapeutic use in RCC in treating patients with either established or high risk of developing metastatic disease.^31^ We therefore sought to characterize the immune environment of SDH-deficient RCCs through flow cytometry, IHC, and analysis of RNA expression profiles.

**Figure 3:**
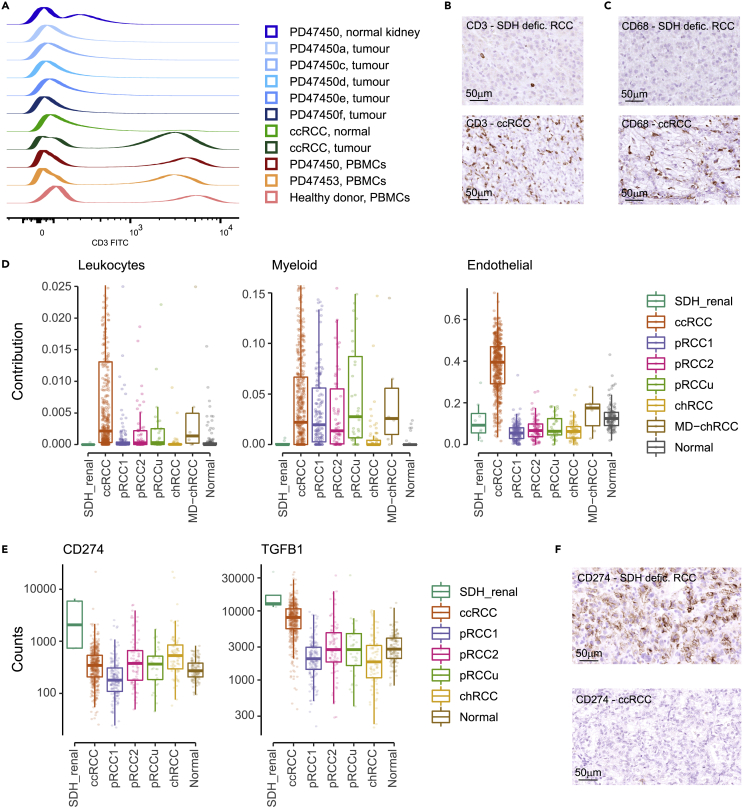
Tumor microenvironment (A) CD3 expression profile of tissue-infiltrating cells extracted from SDH-deficient RCCs, ccRCC, normal kidney, using PBMCs as positive control for detection of CD3-positive T cells (gated on live, single cells). (B and C) (B) Staining of tissue sections by ICH with antibodies specific for CD3 and (C) CD68. (D) Contribution of single cell reference signals of normal cell types^21^ to the range of RCCs (non SDH deficient RCCs from TCGA^26^). Plotted are data points for each sample, alongside the median, first and third quartile, and 95% confidence interval of median. (E) RNA counts of single genes compared to pan-RCC data from TCGA (CD274 log2 fold change 4.4, adjusted p value 4.1×10-16, TGFB1 log2 fold change 0.9, and adjusted p value 0.06). Plotted are data points for each sample, alongside the median, first and third quartile, and 95% confidence interval of median. (F) IHC for CD274 (PD-L1) expression.

Firstly, flow cytometry using both surface and intracellular markers demonstrated SDH-deficient RCCs have low T cell infiltration in comparison to ccRCCs (Figure 3A). These findings were confirmed with IHC, where in addition to an absence of T cells (Figure 3B), there was a scarcity of other immune subsets including macrophages (Figure 3C). The relative scarcity of leukocyte and myeloid-derived immune cells types compared to all other RCCs except chRCC was further confirmed through inferring their presence by deconvolution of the bulk expression profiles using single-cell expression data^32^ (Figure 3D, p< 0.05 by Wilcoxon rank-sum test adjusted by Benjamini-Hochberg).

We next sought to discover the possible mechanisms that could underlie the cold immune tumor microenvironment. Alongside PAPPA2, CD274 (PD-L1) and TGFB1 were both significantly upregulated in our cohort compared to other RCCs (Figure 3E and Table S4). PD-L1, encoded by CD274, is a major co-inhibitory checkpoint signaling ligand that controls T cell activities,^33^ with increased protein expression in SDH-deficient RCCs confirmed with IHC (Figure 3F). Inhibition of this ligand-receptor pathway has consistently shown antitumor effects across multiple tumor types, including ccRCC.^34^ TGFB1 encodes for one of the ligands of the transforming growth factor β protein family, which is known to inhibit host immunosurveillance and exert systemic immune suppression, and is known to play a role in tumor immune evasion and poor responses to cancer immunotherapy.^35^

We finally compared the endothelial contribution to overall RNA expression between SDH-deficient and other RCCs (Figure 3D, right panel). A similar contribution was seen between SDH-deficient RCCs and other non-clear cell RCCs, including normal kidneys (p< 0.05 by Wilcoxon rank-sum test adjusted by Benjamini-Hochberg). As expected, ccRCC showed the highest endothelial contribution reflecting neo-vascularization via the overexpression of VEGF by hypoxia-inducible factor accumulation due to VHL inactivation.^36^ Compared to ccRCC, VEGF-targeted therapy has limited activity on non-ccRCC.^37^

## Discussion

We used well-established analytic pipelines and performed an in-depth profile of the molecular background of SDH-deficient RCCs. We observed a neutral evolutionary trajectory; a kidney epithelial cell of origin, likely within the macula densa; a cold immune pattern with upregulation of PDL1 and TGFB1; and high and specific expression of a potential diagnostic and therapeutic marker, PAPPA2.

In this data, loss of 1p is the second hit event after germline loss of one SDHB allele. The event happens very early in childhood, and is sufficient for carcinogenesis. The mutation burden associated with these tumors is relatively low, is acquired in a time-dependant manner, is notable for the absence of positive selection through secondary driver events, and presents a high degree of subclonality.

Transcriptomic analyses show that the cell of origin for SDH-deficient RCCs may be either macula densa cells, or a precursor to the loop of Henle/distal tubule cells. The uncertainty is in part due to the lack of a clear transcriptomic reference for human macula densa cells, which are a small group of roughly 20 cells in the distal nephron, sitting between the loop of Henle and the distal tubule cells.^28^ The macula densa, a putative neuroepithelial cell, has neuroendocrine functions; sensing the amount of sodium and water that is filtered, releasing autocrine and paracrine signals to the glomerular structures to regulate blood pressure and the amount of blood that is filtered by the renal glomerulus. Patients with inherited SDHB mutations are also exposed to increased susceptibility to neural crest tumors derived from the neuroendocrine chromaffin cells. Given the endocrine parallels between these tumor types and their similar genomic landscape,^18^ it is likely that the acquired genomic alterations may carry similar selective advantages and routes to tumorigenesis. The relative scarcity of the macula densa cells may in part explain epidemiological differences between SDH-deficient RCC and ccRCC, including the rarity of sporadic cases,^8^ and the low penetrance of inherited SDH-deficient RCCs.^10^

One transcript that appears to have been retained from the cellular origin, PAPPA2, was explored as a possible diagnostic aid through the specific use of immunohistochemistry. At present, the clinical diagnosis of SDH-deficient RCC is made through clinical history, microscopic morphological pattern, and absent immunohistochemical staining for SDHB.^13^ These features are not always conclusive. In particular, loss of SDHB expression is not pathognomonic of SDH-deficient RCC. Absent SDHB staining has been described in TSC-associated RCC^38^ and weak staining, albeit not absent, in ccRCC.^39^ Indeed, one of the putative SDH-deficient RCCs in this cohort was, after further analysis, reclassified as a ccRCC. Here, we show that after further diagnostic validation studies, positive PAPPA2 staining can be used as an adjunct to the current diagnostic tests to aid in diagnosis, similarly to the use of stains for 2-succinocysteine alongside fumarate hydratase in hereditary leiomyomatosis and RCC-associated tumors.^40^

Improving diagnostic accuracy is highly relevant not only to adequately inform and refer to patients for germline genetic profiling but also due to important therapeutic implications. In rare diseases, coordinated investigations into systemic therapy are challenging. With increasing possible treatment strategies, it is ever more important therefore to infer cancer susceptibility from molecular and translational studies. In contrast to ccRCC, we observed low endothelial RNA expression contribution in SDH-deficient RCCs, which is consistent with the previously observed poor response to anti-VEGF inhibitors.^14^

We also show a striking lack of immune cell infiltration and provide insights into the possible mechanisms that may drive T cell exclusion. As discussed above, high expression of PAPPA2 is a hallmark in these tumors. Recent work has shown that high levels of pregnancy-associated plasmaprotein-A (PAPPA), a proteinase with similar function and 45% sequence homology to PAPPA2, can be found in Ewing sarcoma, and knockout experiments demonstrated that PAPPA increases the levels of IGF, driving tumor cell proliferation.^41^ Furthermore, PAPPA also inhibited the expression of interferon responsive genes and of HLA molecules required for antigen presentation, thus reducing the immunogenicity of tumor cells. In addition to the postulated immune modulatory effect PAPPA2, SDH-deficient RCCs also employ two further pathways to impair T cell immunity. First, TGF-beta has been identified as a “master regulator” of the tumor microenvironment, capable of increasing the density of the peritumoral extracellular matrix, reducing T cell infiltration.^42^ Second, SDH-deficient RCCs also express high levels of PD-L1, a potent negative regulator of T cell function. Together, the combined effects of PAPPA2, TGF-β, and PD-L1 may explain the observed exclusion of T cells in these tumors, where traditional immune checkpoint inhibitors alone may fail. These observations also provide a rationale for tailored combination therapy targeting PAPPA/IGF-R, TGF-β, and PD1/PD-L1. Targeting PAPPA2, either via T cell engineering^43^or direct binding of anticancer drugs, potentially in combination with checkpoint inhibition therapy, could present as the optimal therapeutic approach for patients who recur after localized disease management or present with upfront metastatic disease.

### Limitations of the study

A larger validation cohort of this rare tumor would be desirable to determine the applicability of findings to high risk disease and the true clinical diagnostic utility of PAPPA2. Any cell of origin inferences rely upon both retained transcriptional signals and an appropriate transcriptomic reference of the macula densa, which has not yet been characterized in humans. Therefore, there remains some uncertainty as to whether the macula densa represents the cell of origin.

## STAR★Methods

### Key resources table

**Table:** 

REAGENT or RESOURCE	SOURCE	IDENTIFIER
**Antibodies**		

anti-CD3 FITC, dilution 1:80, clone SK7	BD Biosciences	#555339
rabbit anti-PAPPA2 (target retrieval solution citrate pH6, dilution 1:30, polyclonal)	Atlas antibodies	#HPA018412; RRID:AB_1854962
rabbit anti-CD3 (target retrieval solution pH9, dilution 1:200, polyclonal	Agilent Dako	#A0452; RRID:AB_2335677
rabbit anti-CD68 (target retrieval solution pH9, dilution 1:100, polyclonal	Invitrogen	#PA5-32330; RRID:AB_2549801
rabbit anti-PDL1 (target retrieval solution pH9, dilution 1:100, clone E1L3N	Cell Signaling	#13684; RRID:AB_2687655

**Chemicals, peptides, and recombinant proteins**		

Collagenasetype IV at 0.01%	Sigma Aldrich	#C4-BIOC
DNAase I at 0.0001%	Roche	#04716728001
Lympholyte-H cell separation media	VHBio	#CL5016
20% dimethyl sulfoxide	Sigma Aldrich	#20-139
Foxp3/Transcription Factor Staining Buffer Set	eBioscience	#00-5523-00
Zombie NIR fixable viability kit	Biolegend	#23106

**Deposited data**		

Whole-genome sequencing data	https://ega-archive.org/	EGAD00001008469
RNA sequencingdata	https://ega-archive.org/	EGAD00001008470
RNA count data	Mendeley Data	https://doi.org/10.17632/ysmbcgyscx.1

**Software and algorithms**		

CaVEMan 1.14.1	Jones et al.^44^	github.com/cancerit/CaVEMan
Pindel 3.3.0	Raine et al.^45^	github.com/cancerit/cgpPindel
BRASS 6.3.0	Campbell et al.^46^	github.com/cancerit/BRASS
Battenberg algorithm 3.5.3	Nik-Zainal et al.^47^	github.com/cancerit/cgpBattenberg
AlleleCounter 3.3.1		github.com/cancerit/alleleCount
STAR	Dobin et al.^48^	github.com/alexdobin/STAR
HTseq 0.7.2	Anders et al.^49^	https://pypi.org/project/HTSeq/
DESeq2	Love et al.^50^	https://doi.org/10.18129/B9.bioc.DESeq2
Cell Signal Analysis	Young et al.^51^	github.com/constantAmateur/cellSignalAnalysis
BayesPrism	Chu et al.^32^	github.com/Danko-Lab/BayesPrism
FlowJo™ v10.8 Software		BD Life Sciences
VAF analysis of SNVs	This paper	https://zenodo.org/record/7162008
Bayes Prism implementation	This paper	https://zenodo.org/record/7162008
Cell signal analysis plotting	This paper	https://zenodo.org/record/7162008
Differential expression analysis	This paper	https://zenodo.org/record/7162008
Timing events in somatic eovolution	This paper	https://zenodo.org/record/7162008
Plotting trinucleotide context	This paper	https://zenodo.org/record/7162008

### Resource availability

#### Lead contact

Further information should be directed to and will be fulfilled by the lead contact, Thomas Mitchell (tjm@sanger.ac.uk).

#### Materials availability

This study did not generate new reagents.

### Experimental model and subject details

All kidney samples were collected from patients enrolled in “Characterisation of the immunological and biological markers of Renal cancer progression” (NHS National Research Ethics Service reference 16/WS/0039) research study. DNA and RNA were extracted from fresh frozen tissue. Formalin-fixed paraffin embedded (FFPE) tissue was used for immunohistochemistry (IHC). Whenever feasible, fresh tissue and peripheral blood samples were collected for flow cytometry. Patient age and gender is detailed in Table S1.

### Method details

#### Mutation calling from whole-genome sequencing

DNA sequencing was performed on the Illumina NovaSeq platform prior to alignment to the GRCh 37d5 reference genome using the Burrows-Wheeler transform (BWA-MEM).^52^ Single base somatic substitutions were called using CaVEMan 1.14.1 (Cancer Variants through Expectation Maximization, https://github.com/cancerit/CaVEMan;^44^). Small insertions and deletions (indels) were called using Pindel 3.3.0 (github.com/cancerit/cgpPindel;^45^). Rearrangements were called using the BRASS 6.3.0 (breakpoint via assembly https://github.com/cancerit/BRASS;^46^) algorithm, which identifies rearrangements by grouping discordant read pairs that point to the same breakpoint event. Copy-number data were derived from whole-genome reads using the Battenberg algorithm 3.5.3 (https://github.com/cancerit/cgpBattenberg;^47^).

#### RNA sequencing

Extracted RNA was isolated and prepared using previously described workflows.^53^ Sequencing was performed on the Illumina HiSeq400 platform. Mapping and QC used the RNA mapping algorithm called STAR [https://github.com/alexdobin/STAR;^48^]. HTseq 0.7.2 was used to generate RNA count data.^49^

#### Flow cytometry

Fresh tissue samples were collected within one hour of surgery and processed into single cells using enzymatic and mechanical dissociation. Briefly, the samples were submerged in a mixture of collagenase type IV (Sigma Aldrich) at 0.01% and DNAase I (Roche) at 0.0001%, and manually sliced using a scalpel. This was followed by three rounds of program h_tumor_01.01 on a gentleMACS dissociator (Miltenyi Biotec) with intervening 30min incubations at 37°C with occasional shaking. Finally, the samples were sequentially filtered using 70μM and 40μM cell strainers, with a final centrifugation step (1800rpm, 10 minutes, RT) followed by resuspension of the pellet, cell counting, and staining.

Peripheral blood mononuclear cells (PBMCs) were isolated from blood using a density gradient medium (Lympholyte-H cell separation media, VHBio), aliquoted with 20% dimethyl sulfoxide (DMSO, Sigma Aldrich), and cryopreserved in vapour phase liquid nitrogen prior to staining.

PBMCs and tissue single cell isolates were stained using two different panels. For the intracellular staining panel, samples were fixed and permeabilised using the Foxp3/Transcription Factor Staining Buffer Set (eBioscience) as per manufacturer instructions.

Samples were run on an LSRFortessa cytometer (BD Biosciences). Instrument performance was assured by running daily cytometer set-up and tracking beads (BD Biosciences). Rainbow calibration particles (BD Biosciences) and software application settings were used to ensure data comparability between samples over time. Compensation was performed using UltraComp beads (eBioscience). Fluorescence-minus-one controls and control PBMCs were used in all experiments.

Data analysis was done using FlowJo™ v10.8 Software (BD Life Sciences). The gating strategy used consisted in selecting the live (Zombie NIR fixable viability kit, Biolegend #23106), single cells and then plotting a histogram against anti-CD3 staining (anti-CD3 FITC, dilution 1:80, clone SK7, BD Biosciences #555339).

#### IHC

For IHC, glass slides with FFPE tissue sections were deparafinised using xylene (Honeywell) followed by sequentially diluted ethanol (Merck) baths. Heat mediated antigen retrieval was done by immersing slides on target retrieval solution (pH9 or citrate pH6, Agilent Dako) and steaming them for 40min using the IHC-Tek epitope retrieval steamer set. Ensuing slide incubations, interspersed by washing steps with a PBS (Oxoid) and Tween 20 (VWR) solution, were done on a humidified chamber in the following order: first with peroxidase-blocking solution (Agilent Dako), followed by primary antibody (60min), and finally with goat anti-rabbit horseradish peroxidase (Agilent Dako). Slides were then incubated with 3,3′-Diaminobenzidine (DAB) substrate-chromogen solution (Agilent Dako) under microscopic vision until suitable development. Incubation with sequentially concentrated ethanol baths ensued, followed by counterstaining with Mayer’s haematoxylin (Sigma Aldrich). After 24h of drying, coverslips were mounted with DPX mounting medium (Merck).

The following anti-human antibodies were used for IHC: rabbit anti-PAPPA2 (target retrieval solution citrate pH6, dilution 1:30, polyclonal, Atlas antibodies #HPA018412), rabbit anti-CD3 (target retrieval solution pH9, dilution 1:200, polyclonal, Agilent Dako #A0452), rabbit anti-CD68 (target retrieval solution pH9, dilution 1:100, polyclonal, Invitrogen #PA532330), and rabbit anti-PDL1 (target retrieval solution pH9, dilution 1:100, clone E1L3N, Cell Signaling #13684).

### Quantification and statistical analysis

#### Estimating mutational burden and subclonality

We used a linear mixed effect model from the R package lme4 to estimate the mutation rate per year. Age was used as a fixed effect with age and donor used as random effects in order to mitigate for multiple samples in one donor. We included an intercept term with the assumption of zero somatic mutations at birth. We recognise that this may either under or over-estimate the true burden, depending on the relative balance of the sensitivity of detecting all mutations present in evolving cancer cells, and the proportion of mutations that are private to multiple subclonal units. Such uncertainties will remain until accurate single cell whole genome sequencing becomes possible. As expected, the mutational burden calculated for the tumour with multi-regional sampling (PD47450) is lower after mutational clustering as it was not possible to place the low cancer cell fraction mutations on the phylogenetic tree.

The subclonality of single base somatic substitutions was interrogated after calculating the cancer cell fraction (CCF). This represents the proportion of cancerous cells in a tumour containing a mutation. We first adjusted the variant allele fraction (VAF) to account for non-tumour cell contamination by dividing by the tumour purity. We then calculated the final CCF by the following:

1. For diploid regions of the genome (as defined by output from the Battenberg algorithm), the adjusted VAF was multiplied by 2.
2. For regions with loss of heterozygosity, the CCF was equal to the adjusted VAF.
3. For regions with a single copy gain (there was no evidence of higher levels of amplification), we first allocated mutations as present either on a single allele, or on the duplicated allele.^16^ The CCF of those mutations on a single allele was calculated as three times the adjusted VAF. The CCF of those mutations on the duplicated allele was calculated as 1.5 times the adjusted VAF.

#### DNA mutational clustering

We tested for true presence or absence of the single nucleotide variants using an approach previously described.^54^ Briefly, counts at each loci were calculated across all samples using AlleleCounter (https://github.com/cancerit/alleleCount). For each patient, the non-tumour samples in this study not belonging to that patient were used as a reference to obtain the locus-specific error rate. To minimize the false positive rate, the presence of the variant in the sample was accepted if the multiple-testing corrected p-value was less than 0.001.

Mutations were clustered using a Bayesian Dirichlet based algorithm as described previously.^19^ Briefly, the expected number of reads for a given mutation present in one allelic copy of 100% of tumour cells may be estimated based upon the ASCAT derived tumour cell fraction, the copy number at that locus, and the total read-depth. The fraction of cells carrying a given mutation is modelled by a Dirichlet process with an adjustment for the decreased sensitivity in identifying mutations in lower tumour fractions. Mutations were thus assigned to clusters according to the calculated fraction of clonality. The hierarchical ordering of these clusters was determined by applying the pigeonhole principle.

#### Timing methods

The timing of chromosome 1p loss was inferred by analysing the number of duplicated and non-duplicated mutations on the isochromosome resulting from the loss of the short arm of chromosome 1, and the additional copy of the long arm of chromosome 1. The emergence of the most-recent common ancestor was also inferred for the multi-region sequenced tumour, PD47450. Methods are as previously published,^16^ although estimates for single region samples could only be estimated directly from patient specific mutational rates, rather than using an estimate of when the most-recent common ancestor occurred. Bootstrapping was used to estimate the 95% confidence intervals. For sample PD47450 there was only one clonal mutation (shared by all regions) on the duplicated arm of chromosome 1. We therefore computed only the timing of the most-recent common ancestor as this emergence is likely to result in the subsequent clonal expansion of the tumour.

#### Differential expression analysis

DESeq2^50^ was used to compute the differential expression of the SDH deficient RCCs. For comparison, the extensive RNA sequencing of RCCs and SDHB deficient pheochromocytoma and paragangliomas from The Cancer Genome Atlas (TCGA) was used,^26^ along-side the clear cell RCC and the SDH deficient paraganglioma sequenced as part of this study. The identification of genes differentially expressed used a combined matrix of raw counts from this study and the TCGA data. The design matrix included the study origin of tissue and whether the samples were SDH deficient. Differential expression was calculated with an FDR threshold of 0.05.

#### Reference RNA single cell expression datasets

To estimate the composition of tumours, and infer the most conserved cellular signals observed in the bulk RNA sequencingdata, we used the following single cell data sets:

(1) An adult kidney reference derived from normal kidneys of patients with RCC.^21^
(2) A fetal kidney reference derived from 16 weeks gestation onwards.^22^
(3) An adult human kidney reference in patients who have RCC with more detailed annotation.^23^
(4) A murine renal single cell dataset, enriched for the distal mouse nephron.^24^
(5) Developing human adrenal tissue.^25^

#### Cell signal analysis and deconvolution from bulk RNA sequencing

We used two methods to analyse the bulk RNA sequencingdata using single cell references. The first method quantifies the sharing of transcripts in bulk tissue with single cell references in order to define better possible tumour cells of origin.^20^ The second method is used to predict cellular composition of the tumours from bulk RNA using single cell references.^32^

In determining possible cells of origin, we compared the bulk RNA to the 5 different single cell references detailed above. We did not include single cell references from cancer cells, as there was no plausible cancer cell of origin, but otherwise included all of the annotated cells in each of the reference sets. To simplify plotting the relative contribution of each single cell reference (Figure 3A), we included only those cell types who mean contribution was at least 2%, and whose contribution was at least 10% in at least one sample. This was done to purely make visualisation easier to the reader, as the most finely annotated reference set contained 118 cell types. The full results table is provided in Table S3. In comparing the contributions across all of the major RCC subtypes incorporating the TCGA data (Figure 3B) we included the adult human kidney epithelial cells^21^ and adrenal single cell reference.^25^ The adrenal reference provided a positive control for cell of origin for the TCGA pheochromocytoma and paraganglioma data,^18^ and provided a sanity check that there was indeed an epithelial cell of origin for the SDH deficient RCCs.

To predict the cellular composition of tumours we collated the reference^21^ cell according to their major lineage (such as leukocytes, myeloid, endothelial, epithelial, fibroblast), rather than use the finely annotated subtypes. The collation to major lineage types helped alleviate batch effects and the difficulty in discerning the presence of subtly different cell subtypes from bulk RNA data. We compared cellular composition of the SDH deficient RCCs with common RCC subtypes from TCGA as described above. Significant differences between contributions from major cell lineages was determined using Wilcoxon rank sum test adjusted by Benjamini-Hochberg.

## Data Availability

Sequence data reported in this paper is European Genome-Phenome Archive: EGAD00001008469 for whole-genome sequencing data, and EGAD00001008470 for the RNA sequencingdata. Raw RNA count data is deposited on Mendeley at 10.17632/ysmbcgyscx.1. All original code has been deposited at zenodo and is publicly available as of the date of publication. DOIs are listed in the key resources table. Any additional information required to reanalyse the data reported in this work paper is available from the lead contact upon request.

## Supplemental information

Document S1. Figures S1–S5

Table S1. Individual clinical data, germline variant annotation and summary sequencing data, related to Figure 1

Table S2. Somatic mutations called across all samples, related to Figure 1

Table S3. Cellular contributions to bulk RNA sequencing results, related to Figure 2A and 3D

Table S4. DE genes between SDH deficient RCCs and the TCGA cohort, related to Figure 2C and 3E
