# Supplementary material for: Defining the origin, evolution, and immune composition of SDH-deficient renal cell carcinoma
Source: iScience. 2022 Oct 17;25(11):105389. doi: 10.1016/j.isci.2022.105389 (PMC9636038; doi:10.1016/j.isci.2022.105389)
Supplement: Document S1. Figures S1–S5 [file mmc1.pdf]

iScience, Volume *25*

Supplemental information

Defining the origin, evolution, and immune composition of SDH-deficient renal cell carcinoma

Joana B. Neves, Kirsty Roberts, Janani Sivakumaran Nguyen, Soha El Sheikh, My-Anh Tran-Dang, Catherine Horsfield, Faiz Mumtaz, Peter Campbell, Hans Stauss, Maxine G.B. Tran, and Thomas Mitchell

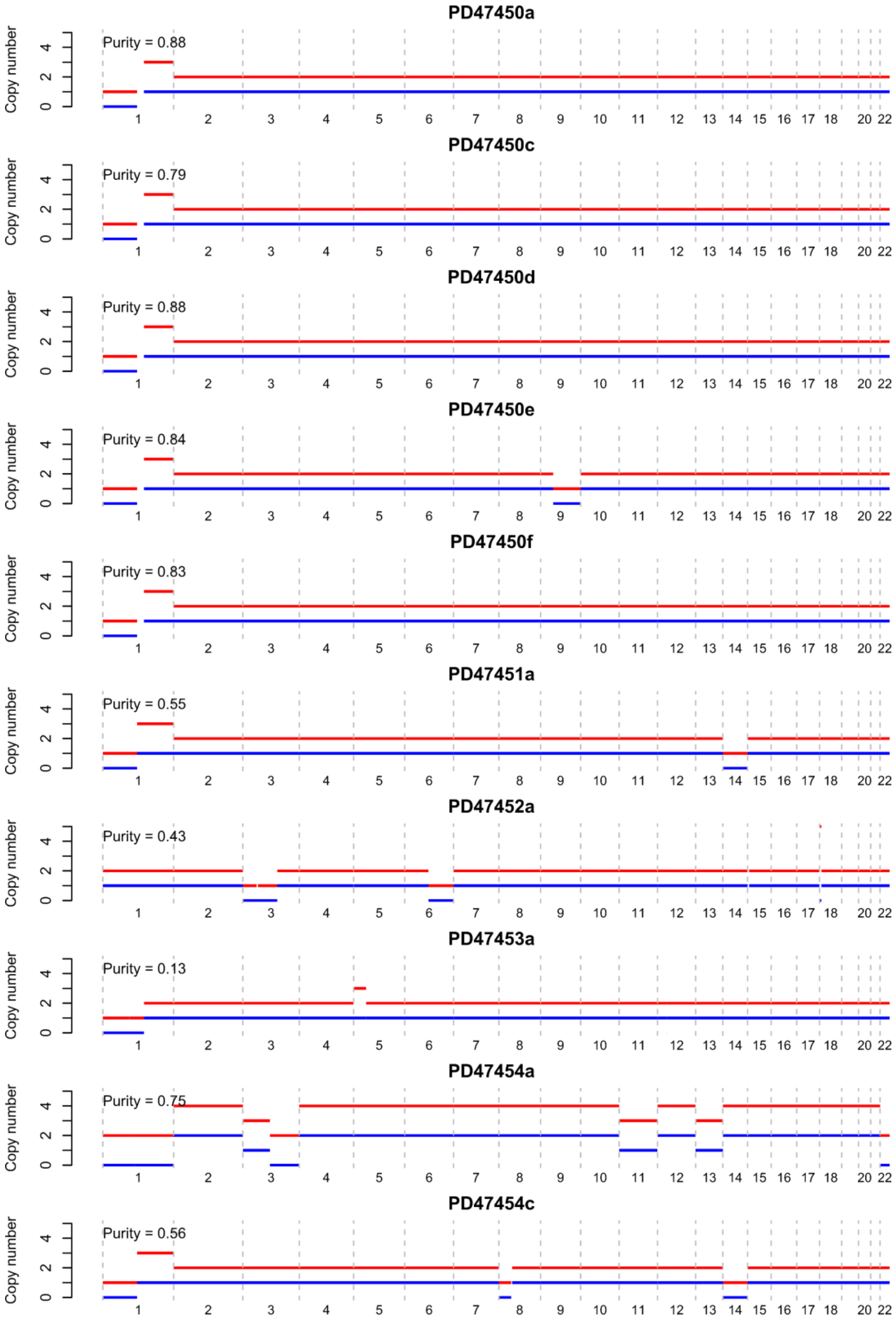

Figure S1. Copy number profiles for all samples, related to Figure 1A. The total (red) and minor allelic (blue) copy number is plotted across the genome for every sample sequenced in this study. PD47450a to f refer to multiregional samples from the same SDH deficient RCC. PD47451a, PD47453a, and

PD47454c refer to samples from three SDH deficient RCCs. PD47452a refers to a sample reclassified as ccRCC. PD47454a refers to SDH deficient paraganglioma from the same patient donating the RCC sample PD47454c.

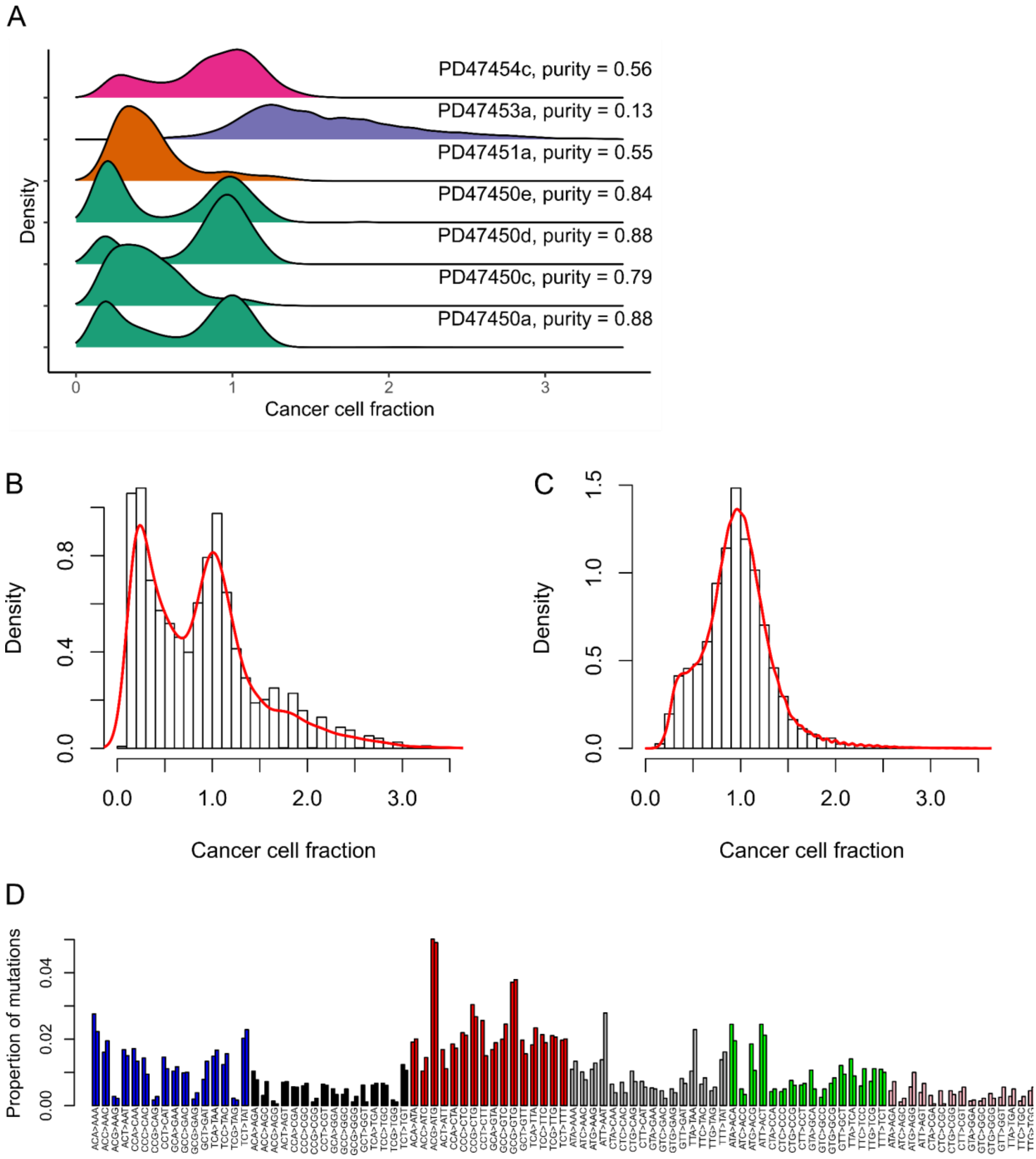

Figure S2. Mutational cancer cell fractions, related to Figure 1C. (A) Density plots of the cancer cell fractions for all SDH deficient RCC samples sequenced. (B) Histogram and three components of mixture model for the cancer cell fraction of all mutations detected in SDH deficient RCCs. (C) As per (B) but plotted for ccRCCs. (D) Trinucleotide context for all mutations called across all SDH deficient RCCs. Left-hand bars denotes clonal mutations with a cancer cell fraction greater than 0.5, the right hand bars denote subclonal mutations with cancer cell fractions less than 0.5.

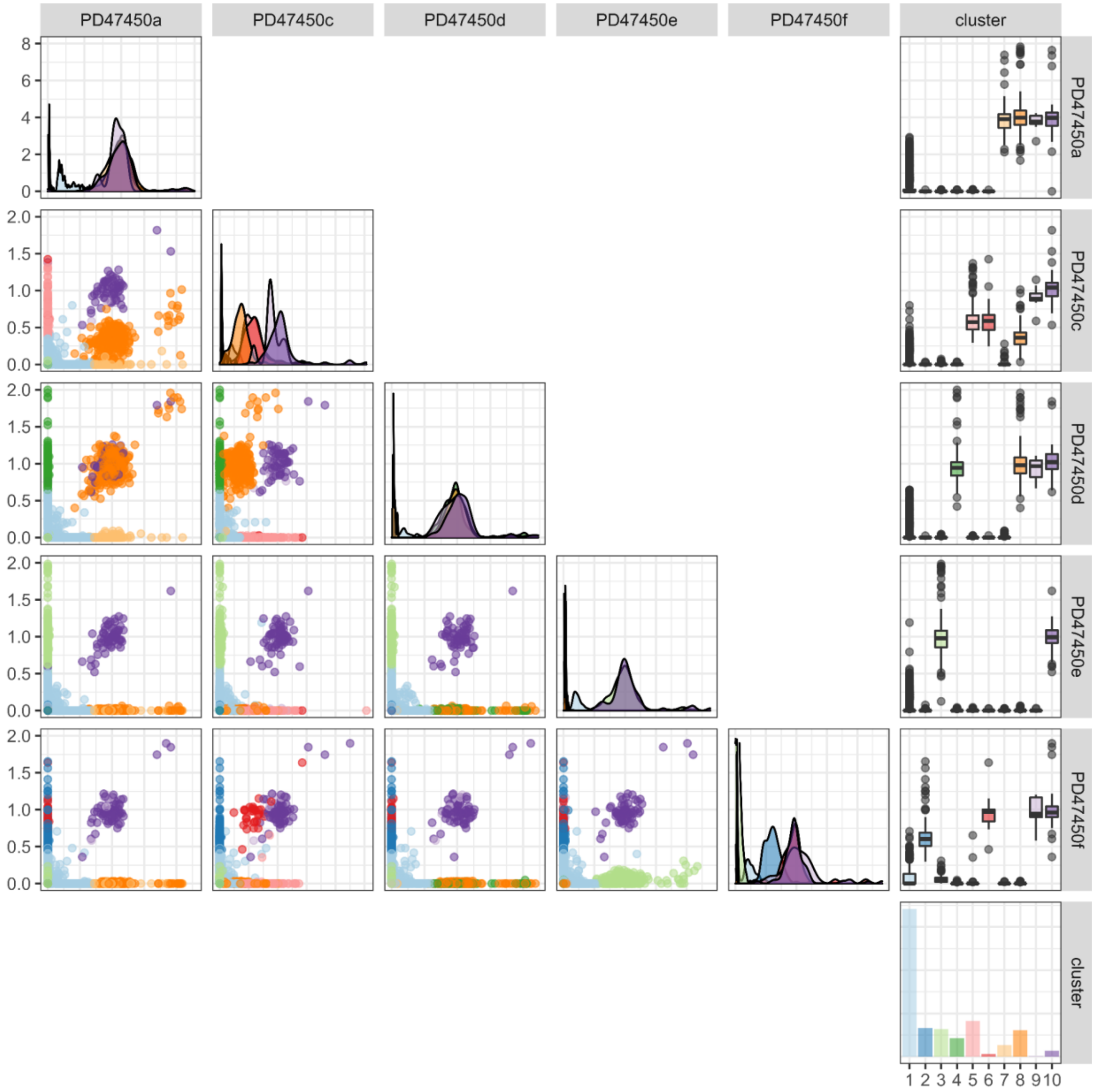

Figure S3. Clustering of mutations from PD47450, related to Figure 1C. Diagonal; density plots of assigned mutational clusters per region. Below diagonal; cancer cell fraction for each mutation, coloured by assigned cluster for each pairwise region. Right side; Median, 95% confidence interval, and outlier cancer cell fractions for each cluster in each sample. Bottom right; Number of mutations in in each cluster. Colours are consistent in each subplot.

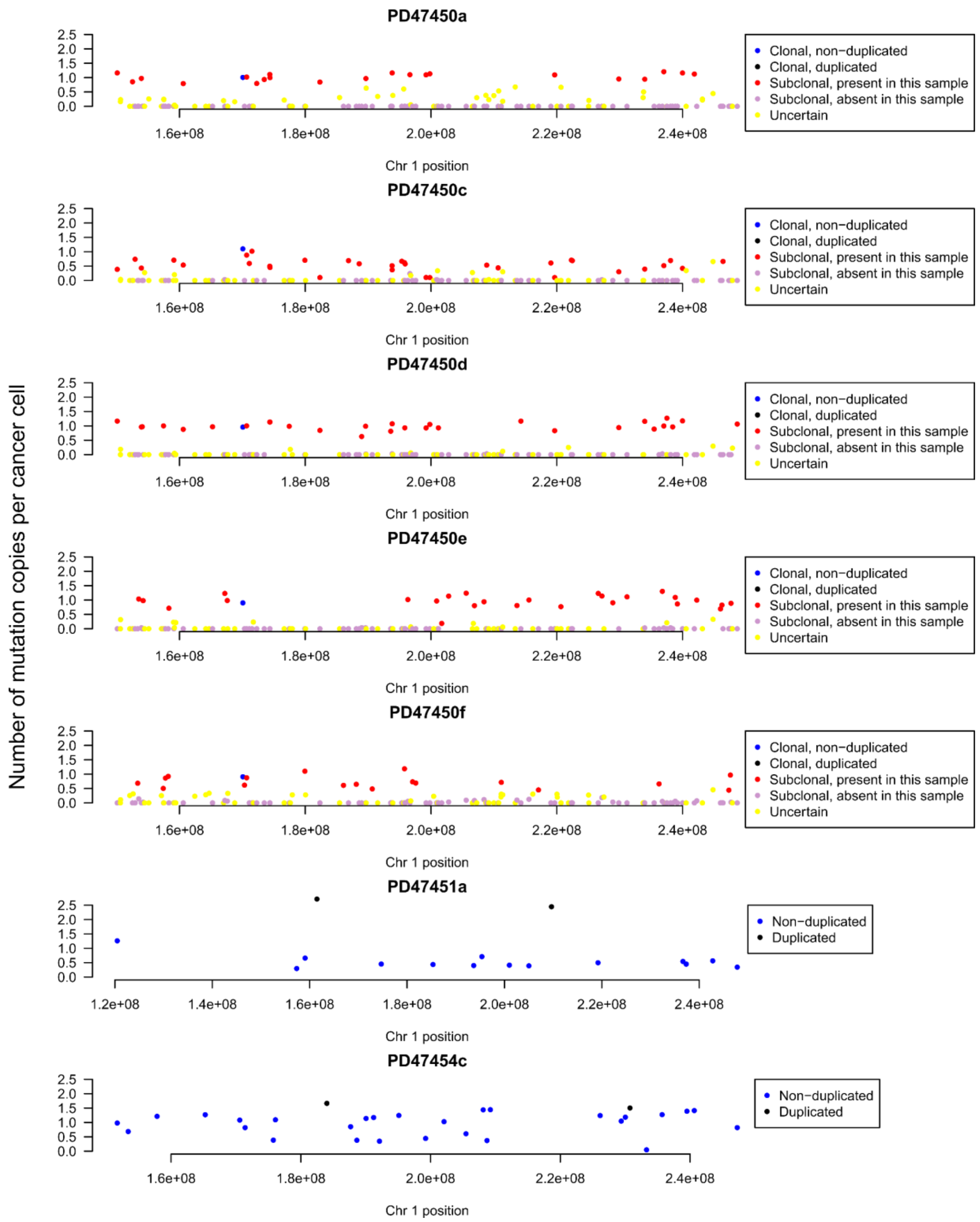

Figure S4. Duplication status of mutations, related to Figure 1D. The cancer cell fraction of mutations on the duplicated chromosome, assigned by duplication status and clonality (where multi-regional sequence data available)

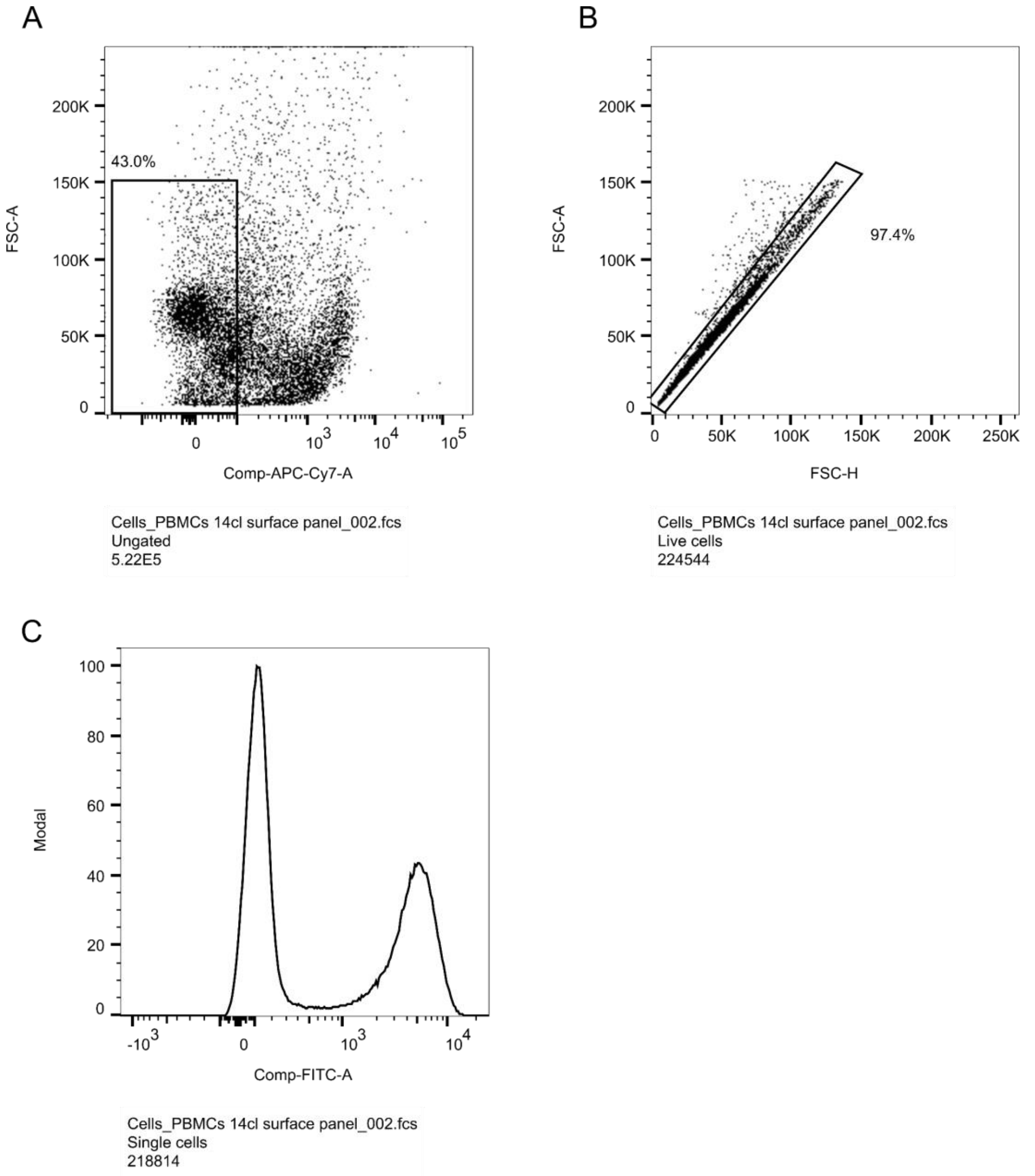

Figure S5. Gating strategy in flow cytometry, related to Figure 3A. (A) Forward scatter area (FSC-A) against Zombie NIR fixable viability stain on the APC-Cy7 channel to select for live cells. (B) Single cells selected by selecting forward scatter area against forward scatter height. (C) Histogram of anti-CD3 on the FITC channel.
